# Supplementary material for: Clinical Features and Outcomes of Acute Kidney Injury in Critically Ill COVID-19 Patients: A Retrospective Observational Study
Source: J Clin Med. 2023 Aug 4;12(15):5127. doi: 10.3390/jcm12155127 (PMC10419665; doi:10.3390/jcm12155127)
Supplement: Supplementary file 1 [file jcm-12-05127-s001.zip › jcm-2204651-supplementary.pdf]

The STROBE statement–Checklist of items that should be included in reports of cohort studies		
Item No.	Item description	Page
Title and abstract		
1a	Indicate the study's design with a commonly used term in the title or the abstract.	1, 4
1b	Provide in the abstract an informative and balanced summary of what was carried out and what was found.	4
Introduction		
2	Explains the scientific background and rationale for the investigation being reported.	6
3	States specific objectives, including any pre-specified hypotheses.	6
Methods		
4	Present key elements of study design early in the paper.	7
5	Describe the setting, locations, and relevant dates, including periods of recruitment, exposure, follow-up, and data collection.	7
6a	Give the eligibility criteria, and the sources and methods of selection of participants. Describe methods of follow-up.	7
6b	For matched studies, give matching criteria and number of exposed and unexposed.	N/A
7	Clearly define all outcomes, exposures, predictors, potential confounders, and effect modifiers. Give diagnostic criteria, if applicable.	7, 8
8	For each variable of interest, give sources of data and details of methods of assessment (measurement). Describe comparability of assessment methods if there is more than one group.	7, 8
9	Describe any efforts to address potential sources of bias.	8
10	Explain how the study size was arrived at.	Figure 1
11	Explain how quantitative variables were handled in the analyses. If applicable, describe which groupings were chosen and why.	8, 9
12a	Describe all statistical methods, including those used to control for confounding.	9
12b	Describe any methods used to examine subgroups and interactions.	8, 9
12c	Explain how missing data were addressed.	8, 16
12d	If applicable, explain how loss to follow-up was addressed.	N/A
12e	Describe any sensitivity analyses.	9
Results		
13a	Report numbers of individuals at each stage of study, e.g., numbers potentially eligible, examined for eligibility, confirmed eligible, included in the study, completing follow-up, and analyzed.	10
13b	Give reasons for non-participation at each stage.	N/A
13c	Consider use of a flow diagram.	Figure 1
14a	Give characteristics of study participants (e.g., demographic, clinical, social) and information on exposures and potential confounders.	10<br>Table 1
14b	Indicate number of participants with missing data for each variable of interest.	Table 1
14c	Summarize follow-up time (e.g., average and total amount).	10
15	Report numbers of outcome events or summary measures over time.	11<br>Figure 2, 3<br>Table 1, 2
16a	Give unadjusted estimates and, if applicable, confounder-adjusted estimates and their precision (e.g., 95% confidence interval). Make clear which confounders were adjusted for and why they were included.	N/A
16b	Report category boundaries when continuous variables were categorized.	N/A
16c	If relevant, consider translating estimates of relative risk into absolute risk for a meaningful time period.	N/A
17	Report other analyses carried out—e.g., analyses of subgroups and interactions, and sensitivity analyses.	N/A
Discussion		
18	Summarizes key results with reference to study objectives.	12
19	Discusses limitations of the study, taking into account sources of potential bias or imprecision. Discuss both direction and magnitude of any potential bias.	15, 16
20	Gives a cautious overall interpretation of results considering objectives, limitations, multiplicity of analyses, results from similar studies, and other relevant evidence.	12, 13
21	Discusses the generalizability (external validity) of the study results.	15, 16
Other information		

22	Gives the source of funding and the role of the funders for the present study and, if applicable, for the original study on which the present article is based.	N/A
